# Uterine Natural Killer Cells: A Rising Star in Human Pregnancy Regulation

**DOI:** 10.3389/fimmu.2022.918550

**Published:** 2022-06-01

**Authors:** Min Xie, Yan Li, Yi-Zi Meng, Peng Xu, Yong-Guang Yang, Shuai Dong, Jin He, Zheng Hu

**Affiliations:** ^1^ Key Laboratory of Organ Regeneration & Transplantation of Ministry of Education, Department of Obstetrics and Gynecology, The First Hospital of Jilin University, Changchun, China; ^2^ National-Local Joint Engineering Laboratory of Animal Models for Human Diseases, The First Hospital of Jilin University, Changchun, China; ^3^ International Center of Future Science, Jilin University, Changchun, China

**Keywords:** uterine natural killer cell, uterine artery remodeling, immune tolerance, pregnancy complications, immunotherapy

## Abstract

Uterine natural killer (uNK) cells are an immune subset located in the uterus. uNK cells have distinct tissue-specific characteristics compared to their counterparts in peripheral blood and lymphoid organs. Based on their location and the pregnancy status of the host, uNK cells are classified as endometrial NK (eNK) cells or decidua NK (dNK) cells. uNK cells are important in protecting the host from pathogen invasion and contribute to a series of physiological processes that affect successful pregnancy, including uterine spiral artery remodeling, fetal development, and immunity tolerance. Abnormal alterations in uNK cell numbers and/or impaired function may cause pregnancy complications, such as recurrent miscarriage, preeclampsia, or even infertility. In this review, we introduce recent advances in human uNK cell research under normal physiological or pathological conditions, and summarize their unique influences on the process of pregnancy complications or uterine diseases. Finally, we propose the potential clinical use of uNK cells as a novel cellular immunotherapeutic approach for reproductive disorders.

## 1 Introduction

The uterus is an important and specialized reproductive organ that can undergo cycles of endometrial shedding and regeneration after puberty in response to fluctuations in sex hormones. Furthermore, the uterus overcomes immune barriers to allow blastocyst implantation and development into the placenta and fetus. Decidualization is specific to the uterus and involves the precise temporal and spatial regulation of multiple factors, including sex hormones, endometrial stromal cells, endometrial stem/progenitor cells, maternal immune cells, and multiple cytokines. Decidualization begins in the second half of menstruation before embryo implantation and expands further after embryo implantation. Spontaneous decidualization can prevent excessive embryo invasion (1), sense embryo quality and reject abnormal embryos (2). This suggests that decidualization is partly responsible for the success or failure of pregnancy. When a blastocyst reaches the uterus, abnormal decidua can lead to implantation failure or abnormal placental and fetal development (3, 4). When the blastocyst does not reach the uterus, decidual tissue dissolves and sheds (menstruation) in response to declining estrogen and progesterone levels. Subsequently, stem cells/progenitor cells in the basal layer regenerate and repair the endometrium and re-epithelialize to form a new functional layer when stimulated by estrogen in the menstrual cycle. The menstrual cycle involves the recruitment of stem cells and renewal of the decidualized endometrium, which ensures that multiple pregnancies do not interfere with each other and maximizes reproductive success (5). Therefore, the success of pregnancy depends not only on the “seeds” but the quality of the “soil”. Decidualization of the endometrium is key to ensuring “soil” quality. uNK cells play an important role in decidualization.

**Figure 1:**
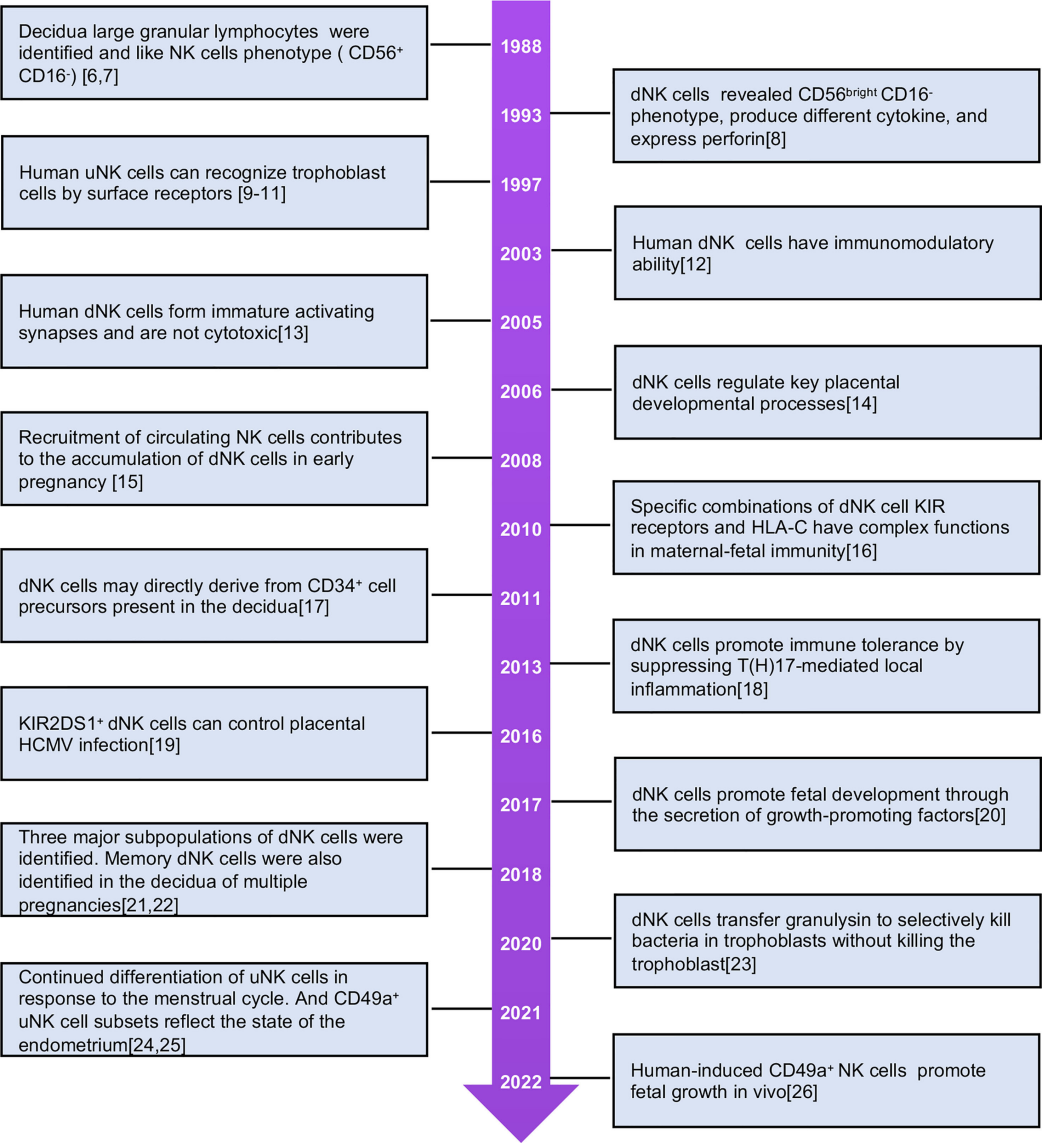
The milestones of human uNK cells investigation.

Research on uNKs began in the 1980s, and many studies have explored their physiological functions (**Figure 1**) (6–26). These cells are abundant in the secretory endometrium and decidua, especially during early pregnancy, accounting for 70% of lymphocytes. uNK cells express tissue-resident receptors, while peripheral blood NK (PBNK) cells do not. Characteristics of tissue-resident NK (trNK) cells in uterine tissues differ from those in non-lymphoid tissues such as the liver, salivary glands, lungs, and fat (27, 28). In the non-pregnant uterus, uNKs in the endometrium, termed eNK cells, are renewed during the menstrual cycle and participate in the decidualization of the endometrium and the reconstruction of blood vessels, creating favorable conditions for embryo implantation. eNK cells can continue to differentiate in response to periodic changes and endometrium regeneration. These cells flow out of the uterus with menstrual blood and are termed menstrual blood NK (MBNK). eNK cells have characteristics similar to dNK cells (21, 24, 29). It is speculated that secretory eNK cells may be a group of immature NK cells waiting for pregnancy (30). When the embryo is implanted, uNK cells proliferate further and differentiate in the decidual environment and express a variety of cytokines. The cells are involved in the formation of uterine arteries and control the growth and development of embryos (31). dNK cells express multiple inhibitory NK cell receptors (iNKRs), including killer cell immunoglobulin-like receptors (KIRs), leukocyte immunoglobulin-like receptors, and C-type lectin-like receptor families (NKG2/CD94). The cells specifically bind to non-classical MHC-I molecules on the surface of trophoblast cells and mediate recognition and immune tolerance functions in the embryo.

Furthermore, multiple cytokines can be secreted by dNK cells, contributing to a successful pregnancy. The altered cytokine secretion profile of dNK cells is associated with multiple pregnancy complications, such as recurrent miscarriage (RM), preeclampsia (PE), and intrauterine fetal growth restriction (FGR) (31). In addition, dNK cells can clear microbial infections without killing trophoblast cells, limit the expansion of inflammation in the placenta, and ensure a successful pregnancy (23). Previous studies have revealed the mechanism by which dNK cells promote successful pregnancy and have emphasized their importance in early pregnancy. In contrast, much less attention has been paid to eNK cells prior to pregnancy.

This review summarizes the characteristics and functions of uNK cells in non-pregnancy and pregnancy and details the possible mechanisms by which uNK cells mediate embryo implantation and placental fetal development. In addition, the mechanism of action of uNK cells in various reproductive diseases is discussed from multiple perspectives. The potential use of uNK cells for these complications is highlighted.

## 2 uNK Cells Under Normal Physiological Conditions

**Figure 2:**
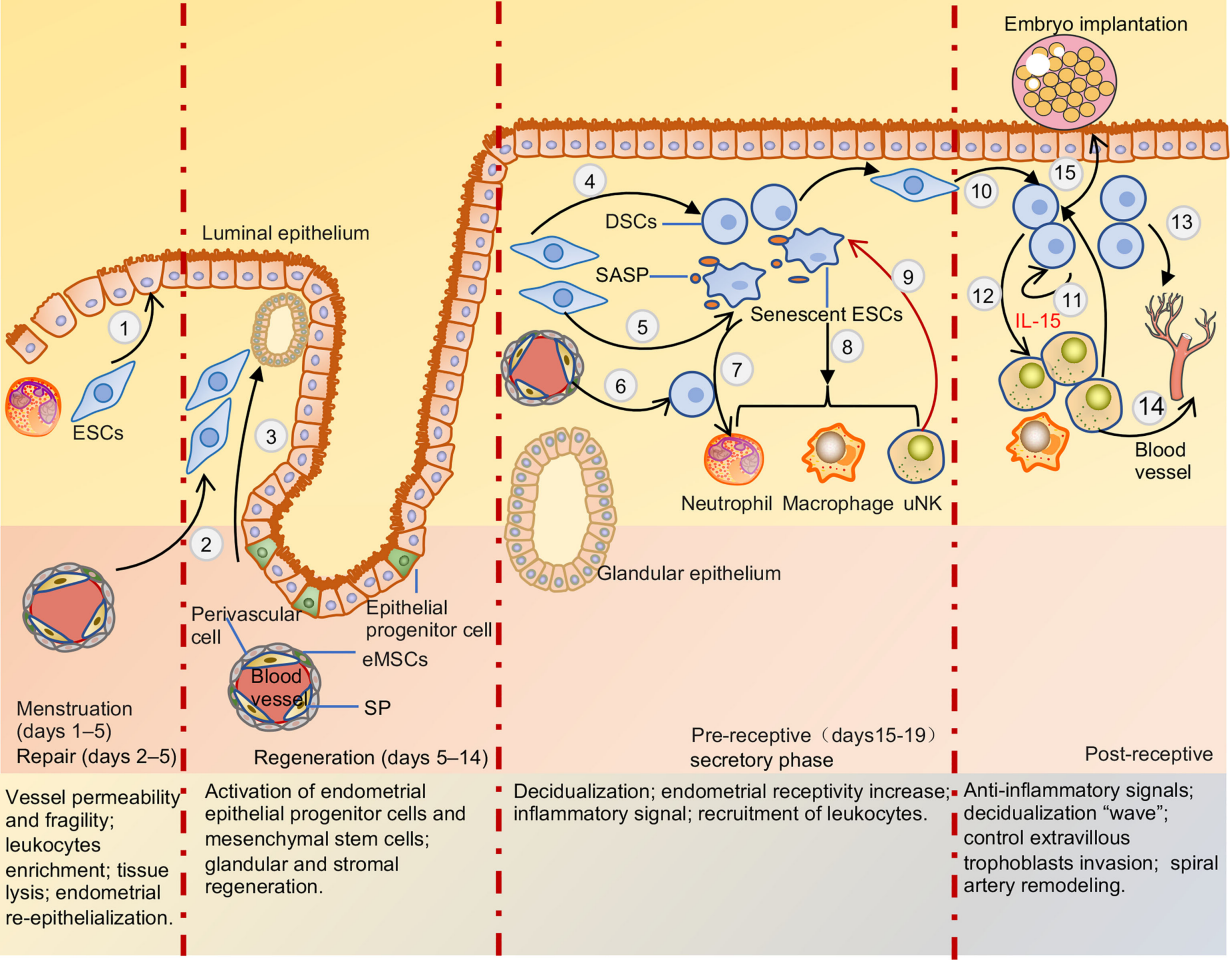
Menstruation cycle and pre-receptive and post-receptive endometrium. When the embryo is not explanted, DSCs secrete multiple chemokines that drive inflammatory cells into endometrial due to hormone withdrawal, recruitment of leukocyte increased activation and production of proteolytic enzymes, enhance reactive oxygen species release, and inhibition of superoxide dismutase activity, leading to tissue destruction and lysis. Post-menstrual repair of endometrium mainly depends on epithelium migration and endometrial stromal cells differentiation (1) to facilitate re-epithelialization and terminate bleeding. Regeneration of the endometrium is mainly mediated by endometrial epithelial progenitor cells and perivascular mesenchymal stem cells differentiation glands and stroma respectively (2,3). After ovulation, endometrial stromal cells and mesenchymal stem cells can be transformed into decidua stromal cells due to the withdrawal of estrogen and progesterone (4,6). Endometrial stromal cells also can be transformed into senescent decidual cells in the process of decidualization (5). Cytokines secreted by decidua stromal cells and SASP released by senescent decidual cells recruited leukocytes into functional layers to maintain a pro-inflammation environment (7,8). uNK cells can clear senescent decidual stromal cells and terminal differentiation of decidual cells, support the conversion of endometrial pro-inflammatory signals to anti-inflammatory signals, and facilitate embryo adhesion (9). After the embryo is implanted, decidual stromal cells generate a ‘wave’ of decidualization by autocrine and paracrine cytokines and spread throughout the uterus (10,11). And decidual stromal cells significantly induce uNK cells proliferation and differentiation by secreting IL-15 (12). Multiple cytokines and angiogenic factors secreted by decidua stroma cells, uNK cells, and macrophage cells induce uterine spiral arteries to remodel (13,14). Meanwhile, uNK cells and decidual stromal cells can control EVT cells invasion and sense embryo quality (15). ESCs, endometrial stromal cells; eMSCs, endometrial mesenchymal stem cells; SP, side-population cells; DSCs, decidua stromal cells; SASP, senescence-associated secretory phenotype; uNK, uterine natural killer cell.

The endometrium is a special multicellular steroid-target tissue that comprises the underlying basal and outer function layers. The functional layers undergo breakdown, repair, regeneration, and decidualization related to the dynamic changes in sex hormones (**Figure 2**). Endometrial decidualization provides a “fertile ground” for blastocyst implantation and subsequent normal placenta formation (4). The quality of this “fertile ground” is affected by a variety of factors, including sex hormones, decidualization of endometrial stromal cells, generation and clearance of senescent cells, chemotaxis of inflammatory cells, and enrichment of eNK cells (**Figure 2**) (32–35). These cells can increase the receptivity of the endometrium, sense embryo quality, and play an important role in embryo implantation (33, 36). When the embryo is implanted, decidua stromal cells (DSCs) create a “wave” of decidualization by local paracrine or autocrine factors (37). DSCs secrete multiple cytokines that support the proliferation and differentiation of dNK cells during early pregnancy. dNK cells play important roles in inducing uterine spiral artery remodeling, fetal and placental development, and resistance to microbial infections. The differences in receptor expression of NK cells in the endometrium, menstrual blood, decidua, and peripheral blood are detailed below. Unlike PBNK cells, NK cells have similar receptor expression profiles in the non-pregnant uterus and decidua during early pregnancy. However, the cytokine secretion profiles of eNK cells differ from those of dNK cells.

### 2.1 Origin of uNK Cells

There is no consensus on the origin of uNK cells. Increasing evidence suggests that uNK cells may have multiple origins. Several studies have shown that uNK cells may be directly derived from CD34(+) cell precursors present in the endometrium or decidua, which differentiate into eNK or dNK cells by interacting with components of the endometrial or decidual microenvironment (17, 38, 39). In addition, one source of uNK cells may be through the recruitment of CD34(-)CD117(+)CD94(-) NK precursor cells in the peripheral blood to the endometrium or decidua, where the cells can differentiate into uNK cells *in vitro* (40). Additionally, PBNK cells may acquire a uNK cell-like phenotype when exposed to a medium conditioned with transforming growth factor-beta (TGF-β) (41, 42). Recent research has demonstrated that uNK cells are transient tissue-resident cells in the endometrium of human leukocyte antigen (HLA)-mismatched uterine transplant patients and can be replenished from the circulation (24). These results indicate that the source of uNK enriched in the endometrium or decidua is probably attributed to the combined effects of the periphery and uterus.

### 2.2 eNK Cells

eNK cells are critical leukocytes that have been intensively studied for over two decades. The number of eNK cells undergoes dynamic changes in the menstrual cycle and reaches the highest level in the secretory phase, accounting for approximately 30% of the total lymphocytes in the endometrium (35, 43–45). eNK cells have a strong proliferative ability in the secretory phase and express the proliferation marker Ki67 (24, 46), which can be shed during menstruation and then recruited to the endometrium from the periphery. They differentiate in response to endometrial regeneration (47).

#### 2.2.1 eNK Cell Phenotype

CD56^bright^ CD16^-^ NK cells are main type of eNK cells, comprising approximately 70% of total NK cells during the secretory and menstrual phases (25). Approximately 90% of PBNK cells are CD56^low^ CD16^bright^ NK cells. eNK cells express multiple tissue residency markers (CD49a, CD9, and CD69) (48); most eNK cells are CD56^bright^ CD16^−^ KIR^+^ CD9^+^ CD49a^+^ phenotype and lack CD16 and CD57 expression. eNK cells express multiple receptors, including KIRs, leukocyte immunoglobulin-like receptor B1 (LILRB1), NKG2A/C/E receptors, and natural cytotoxicity receptors (NCRs). However, their receptor expression profiles differ significantly from those of PBNK cells (**Table 1**). The KIR repertoire of MBNK cells favors KIR2D receptor expression, which is similar at the end of each menstrual cycle. Additionally, menstrual blood NK cells co-expressing two or three KIR receptors are more frequent than peripheral blood NK cells (51). A recent study showed that eNK cells continue to differentiate in response to cyclic changes and regeneration of the endometrium by acquiring CD39 and KIR receptor expression. This differentiation pathway has also been verified in humanized mouse models (24). KIR^+^ CD39^+^ eNK cells appear in the late stage of the menstrual cycle; during pregnancy, the size of the cell population is partially genetically controlled, and they are replenished from the circulation following menstrual outflow. KIR^-^ CD39^-^ eNK cells are present mainly in the early and middle stages of the menstrual cycle. Differentiated eNK cells exhibit reduced proliferative and enhanced pro-angiogenic capacities (24). Interestingly, these CD39^+^KIR^+^ double positive NK cells are mainly detected in uterine and decidual but not in other organs, such as the liver and tonsil (24). In addition, a single-cell sequencing study of decidua in early pregnancy revealed the significant increase of KIR^+^ CD39^+^ uNK cells and their important role, as described in detail below.

**Table 1:** Characterization of human natural killer (NK) cells in endometrial, menstrual blood, decidua, and peripheral blood.

Phenotype	eNK	MBNK	dNK (First trimester)	PBNK
CD49a	+++ (49)	+++ (25)	+++ (25)	- (25)
CD9	+++ (50)	++ (51)	+++ (52, 53)	- (12, 50)
CD103	++ (49)	++ (45)	++ (49)	- (45)
CD69	+++ (54)	++ (45)	++ (53)/+++ (55)	+ (54)/++ (45)
NKG2A	+ (56)	+++ (57)	+++ (53, 56)	++ (57, 58)
NKG2C	+~++ (59)	++ (57)	+~++ (22)	+ (57)
NKG2D	+ (56)	ND	++ (53, 56, 60)/+++ (55, 61)	+++ (62, 63)
KIR2DL1	+~++ (54, 64)	++ (57)	++ (65, 66)	+ (65)/+~++ (54)
KIR3DL1/S1	+~++ (58)	ND	++ (55, 58, 67)	++ (67)
KIR2DL2/L3	+ (64)	ND	+++ (68)	++ (62, 64)
KIR2DL3	ND	++ (57)	++ (65)	+ (57)
KIR2DS4	ND	ND	+ (55)/++ (52)	++ (52)
KIR3DL1	+~++ (58)	+ (57)	+~++ (58, 65)	+ (57, 65)
KIR2DL4	ND	ND	+ (55)/++ (69)	ND
KIR2DL1/S1	ND	++ (57)	++ (52, 55)/+~++ (67)	++ (57, 67)/+ (52)
KIR2DL2/L3/S2	++ (58)	+++ (57)	++ (55, 58, 65, 67)	++ (57, 65, 67)
NKP44	+ (49, 70)	+(45)	+ (49, 60)/++ (55)	- (45)
NKP30	+~+++ (70)	++ (45)	++ (53)/+++ (55, 60)	++ (62, 63)
NKP46	+++ (70)	++ (45)	+++ (53, 60)	++ (45, 63)
LILRB1	+~++ (59)	+++ (57)	++ (53, 55, 65)	++ (57, 65)

(-) No expression; (+) <20%; 20%< (++) < 60%; (+++)>60%; ND (Not Detected); eNK, endometrial natural killer cell; MBNK, menstrual blood natural killer cell; dNK, decidual natural killer cell; PBNK, peripheral blood natural killer cell.

Owing to the limitations of endometrial sampling, there are few studies on eNK cells. In addition, the diversity in the method, site, and time of material collection among multiple experiments leads to diverse results, which complicates the integration of data from multiple studies. MBNK cells may be a good substitute for eNK cells in overcoming these difficulties. MBNK cells are obtained easily and non-invasively, are very similar to eutopic eNK cells and are more stable during each menstrual cycle. Therefore, studies that utilize MBNK cells could clarify the characteristics and physiological functions of eNK cells.

#### 2.2.2 eNK Cell Function

Few studies have examined the physiological functions of human eNK cells. Most of these studies have focused on dNK cells in the first trimester, contributing more to embryo implantation and menstruation. There are three main physiological functions of eNK cells. First, senescent and terminally differentiated decidual cells likely secrete cytokines that lead to recruitment/activation of immune cells in the endometrium that promotes embryo implantation. eNK cells can target and eliminate these cells to convert endometrial pro-inflammatory signals into anti-inflammatory signals to maintain endometrial homeostasis (33). Second, normal embryos secrete higher levels of hyaluronan to enhance eNK cell clearance of decidual senescent cells. In contrast, embryos that fail implantation have been shown to have secreted less hyaluronan leading to inhibition of NK cell activity. These results suggest that eNK cells can determine the fate of an embryo implanted in the endometrium by determining its quality (36). Third, eNK cells can participate in the development of blood vessels in the endometrium and preparation of the endometrium before menstruation by secreting chemokine (C-C motif) ligand 2 (CCL2), which can be directly regulated by estrogen (71–73). Abnormality of eNK cells may be one of the causes of multiple recurrent implantation failures or RM, and may also be responsible for various pregnancy complications caused by dysregulation of dNK cells in early pregnancy. The loss of function or presence of eNK cells before pregnancy may indicate or predict high-risk factors for complications during pregnancy. These possibilities warrant further study.

### 2.3 dNK Cells

**Figure 3:**
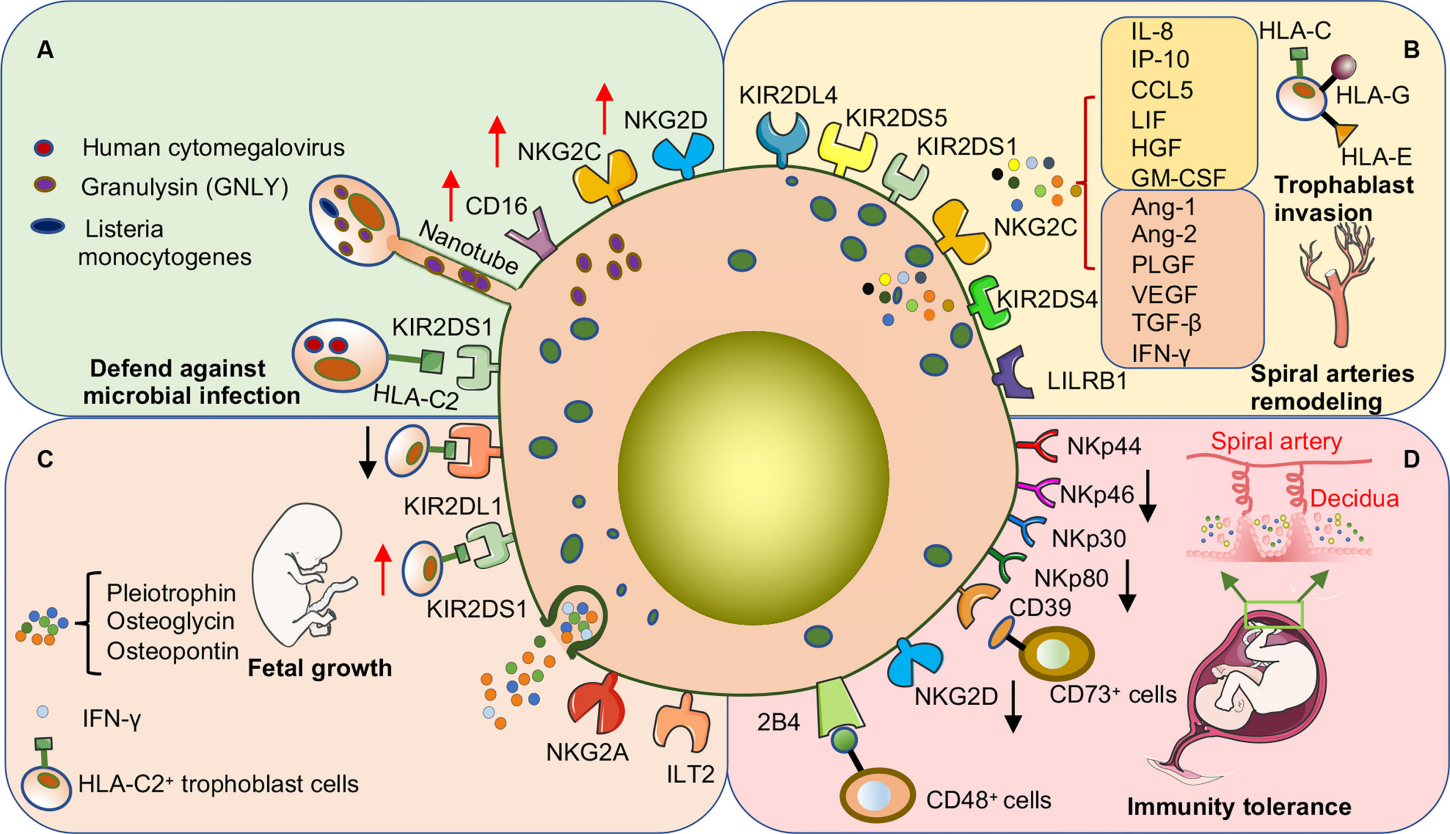
dNK cells promote pregnancy success through surface receptor expression or cytokine secretion in early human pregnancy. **(A)** dNK cells are important in defense against microbial invasion at the maternal-fetal interface. dNK cells deliver GNLY through nanotubes into trophoblast cells infected with *Listeria monocytogenes* to kill the bacteria without damaging the trophoblast cells. In addition, the expression of various activating receptors or the acquisition of CD16 is involved in the process by which dNK cells kill other microbial infections. **(B)** dNK cells promote trophoblast invasion and participate in the remodeling process of uterine spiral arteries. The above functions are induced by the direct binding of activating or inhibitory receptors on the surface of dNK cells to ligands on the surface of trophoblast cells and may also be involved in promoting the secretion of various cytokines. **(C)** dNK cells promote embryonic growth and development by secreting growth-promoting factors. In addition, activation or inhibition of dNK cells severely affects embryonic growth. Co-expression of the activating receptor KIR2DS1 and the inhibitory receptor KIR2DL1 of dNK cells regulates the balance of embryonic growth, especially when the embryo inherits the paternal HLA-C2 gene. **(D)** Decreased expression of various activating receptors is one of the main mechanisms by which dNK cells maintain immune tolerance at the maternal-fetal interface. CD39 is highly expressed on dNK cells, which can bind to CD73 on the surface of epithelial glands and EVT cells to convert ATP to adenosine to maintain immune tolerance.

dNK cells are the most important lymphocytes in the decidua during the first and second trimesters of pregnancy. They account for approximately 70% of the total lymphocytes in these trimesters and approximately 50% in the third trimester (60, 68). In the first trimester, approximately 90% of dNK cells are composed of the CD56^+^ CD16^−^dNK cell subset. The frequency of this subset is significantly reduced in the third trimester (60, 74, 75). These studies also demonstrated the alterations in the receptor profile, degranulation capacity, and cytokine secretion functions of dNK cells during pregnancy (**Table 2**). The characteristics, subpopulations, and functions of dNK cells are modified to better adapt to changes in gestational age and decidual tissue type and to precisely adjust the interaction with EVT cells, allograft recognition, spiral artery remodeling, fetal development, immune tolerance, and defense against microbial infection (**Figure 3**). Failure to properly regulate these changes in dNK cells may be associated with various pregnancy-related complications. However, current research on dNK in the second trimester focuses on pathological pregnancy. The function of dNK cells in a physiological state is poorly understood due to the limitations of clinical samples. Detailed investigations of dNK subsets, their characteristics, and their dynamic function throughout pregnancy are critical to understanding the development of pregnancy complications.

**Table 2:** Phenotypes and function of human decidua NK cells in the first trimester, second trimester, and term trimester.

Frequency of dNK (%)	First trimester	Second trimester	Term trimester
CD56^+^ CD16^−^	95%	95%	85%
KIR2DL1	++	ND	+
KIR2DS1	+	ND	+/-
KIR2DL2/3	+++	ND	+
NKG2D	+	++	++
NKG2A	+++	+++	+++
NKp30	+++	+++	++
NKp46	+++	+++	+++
NKp80	+	++	++
NKp44	+	+	+
CD107a	++	+	+++
IFN-γ	++/+	++	+
TNF-α	+	ND	+

+/- < 10%;10% < + < 30%;30% < ++ < 60%; 60%>+++; ND (Not Detected). [Key references (60, 68, 74–76)].

#### 2.3.1 dNK Cell Phenotype

dNK cells share many phenotypic similarities with eNK and MBNK cells but are significantly different from PBNK cells (**Table 1**). This could be attributed to the temporal and spatial interactions between the uterine microenvironment and dNK cells. For example, the cytotoxicity of dNK cells is weak, although these cells contain more granzymes. Moreover, dNK cells have a strong ability to secrete cytokines and contribute to a successful pregnancy. In addition, unlike the activating isoforms of NCRs (NKp30 and NKp44) expressed by PBNK cells, dNK cells selectively express inhibitory isoforms of NCRs. Many soluble cytokines present in the decidua control the NCR switch (NKp30, NKp44) splice variant profile from PBNK cells to dNK-like cells. This switch contributes to the reduced cytotoxic function and promotes immune tolerance (77). dNK cells also exhibit a unique inhibitor profile that is selectively expressed over time during gestation in the physiological state. This profile is biased to recognize HLA-C expressed by trophoblast cells and is regulated by the local microenvironment of the pregnant uterus (67).

dNK cells express inhibitory receptors, mainly KIRs, NKG2A, and LILRB1. Receptors for KIRs on dNK cells are highly polymorphic and are affected by maternal HLA-C, geographical distribution, and ethnic differences, which have a greater impact on pregnancy outcomes (65, 78–80). The family of KIRs is located on human chromosome 19 and is highly polymorphic. The *KIR* genotype comprises two *KIR* haplotypes, *A* and *B. KIR A* haplotypes consist of seven genes, whereas *KIR B* haplotypes possess 12 genes. In addition, KIR can be designated based on the number of extracellular immunoglobulin-like domains (2D or 3D) and the length of the cytoplasmic tail (L for long and S for short) (81). *KIR A* haplotypes mainly feature inhibiting receptors, including KIR2DL1/3, and potential activating receptor KIR2DS4. *KIR B* haplotypes mainly include KIR2DL1/2 and activating receptor KIR2DS1 (82). The *KIR* genotype of the pregnant mother is *AA* or *AB/BB*. Maternal *HLA-C* alleles regulate the response of dNK cells by altering the frequency of the homologous receptor KIRs. For example, dNK cells isolated from women carrying the *C2* epitope of the HLA-C allele feature reduced expression of the homologous receptor KIR2DL1. However, KIR2DL3 receptor expression is increased in dNK cells with the *C1* epitope. Interestingly, PBNK cells do not exhibit these changes under the same conditions (65). In addition, due to the diversity of maternal *KIR* genotypes and fetal trophoblast *HLA-C*, which affects the physiological function of dNK cells during pregnancy to promote placental development, some specific genotypes may cause pathologically related pregnancy complications (83). Moreover, unlike PBNK cells, dNK cells highly express the inhibitory receptor NKG2A, which enables NK cells to function and bind to the non-classical MHC class I molecule HLA-E on the trophoblast cell surface. LILRB1, which contains an immunoreceptor tyrosine-based switch motif expressed by approximately 40% of dNK cells, is an inhibitory receptor for all HLA class I molecules. However, LILRB1 has a stronger affinity for the HLA-G dimerized form on EVT and acts as an activating receptor (84). Different NK cell types can express distinct expression patterns of receptors. Nearly half of dNK cells can express three or more inhibitory NK cell receptors (iNKRs); however, <10% of PBNK cells do likewise. With the increase in iNKRs, the proliferative capacity of dNK cells is also enhanced by elevated sensitivity to interleukin (IL)-15. In addition, the interaction of maternal HLA-C with KIR2DL1 causes dNK cells to gain function, which is enhanced by the NKG2A receptor. Interestingly, the expression of LILRB1 downregulates the responses of the dNK cell subpopulation and overcomes any educational effect of KIR2DL1 or NKG2A, which is significantly different from PBNK cells (65). Because, compared with PBNK cells, dNK cells have different phenotypes and receptor expression profiles, the interaction between the inhibitory receptors on its surface and corresponding ligands leads to different immune modulating effects between dNK cells and PBNK cells.

**Figure 4:**
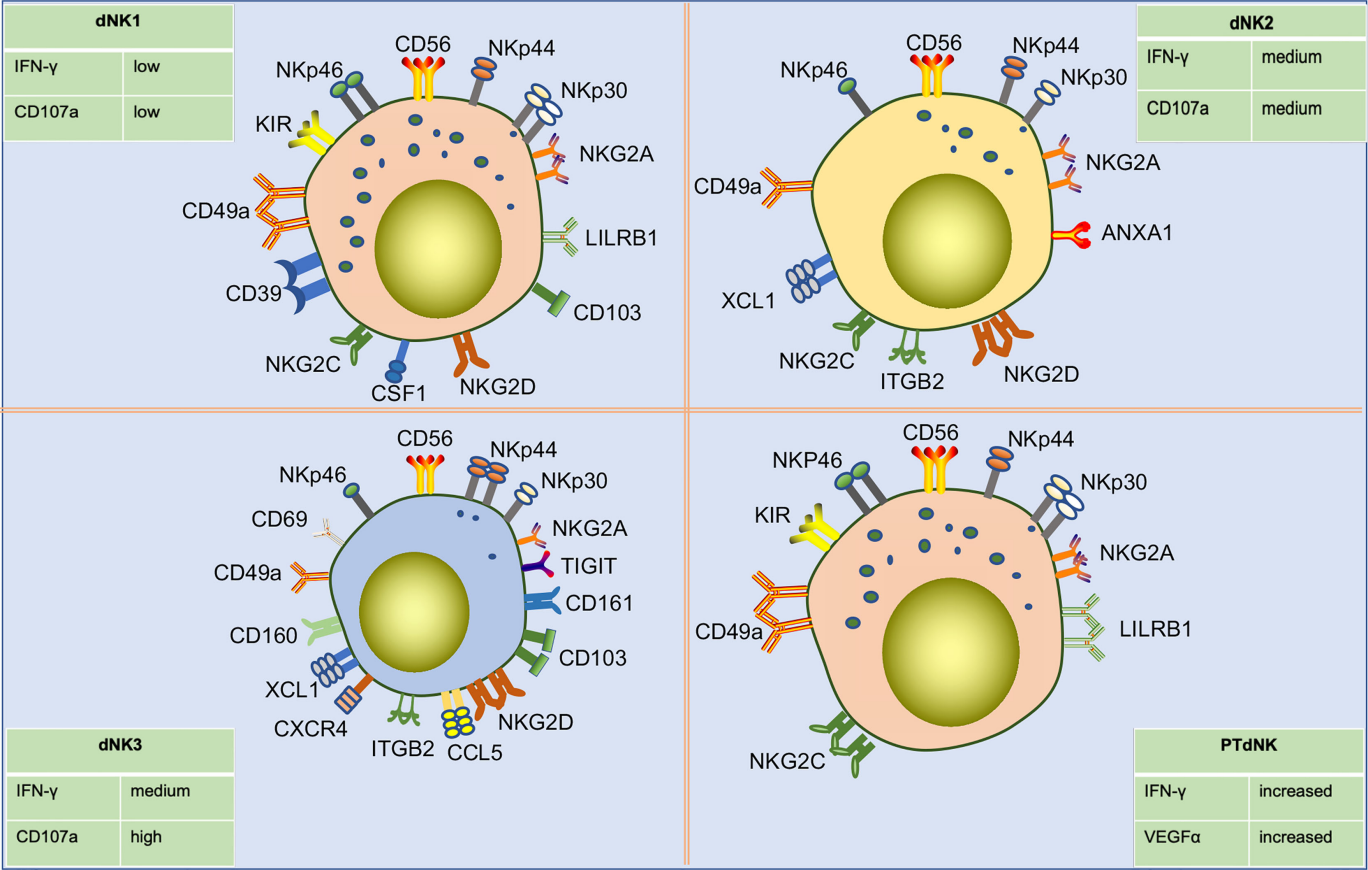
Subpopulations of human dNK cells in early pregnancy and their differences in surface receptor expression profiles. Single-cell sequencing showed that dNK cells in early pregnancy were divided into three subpopulations, including dNK1, dNK2, and dNK3. dNK1 cells are CD56^bright^ CD49a^high^ KIR^high^ NKG2A^high^ LILRRB1^high^ CD39^high^ phenotype, about 55% of total dNK cells. dNK2 cells are CD56^bright^ CD49a^+^ KIR^low^ NKG2A^high^ LILRRB^-^ CD103^-^ phenotype, about 15% of total dNK cells. dNK3 cells are CD49a^+^ CD69^high^ CD103^high^ CD161^high^ KIR^low^ NKG2A^low^ NKP44^+^ NKG2D^high^ phenotype, about 15% of total dNK cells. Additionally, three dNK types have different degranulation capacity and IFN-γ secretion capacities in response to nonspecific stimulation. Pregnancy-trained memory NK cells (PTdNK) are found in the decidua of multiple pregnancies, like dNK1, with CD56^bright^ KIR^high^ NKG2A^high^ LILRRB1^high^ NKG2C^high^ phenotype. In the presence of IL-15, the interaction of NKG2C with HLA-E and LILRB1 with HLA-G induced increased amounts of IFN-γ and VEGFa secreted by PTdNK. And *IFNG* and *VEGFA* were found to have increased accessibility in PTdNK cells by ATAC-seq analysis.

Using high dimensional mass cytometry (cytometry by time of flight, CyTOF), Colucci and Sharkey’s group identified three sub-phenotypes of early gestational dNK cells as dNK1, dNK2, and dNK3 based on surface markers and receptor expression (85)(**Figure 4**). dNK1 cells predominate. These cells specifically express LILRB1 and highly express multiple KIR. These findings indicate that dNK1 cells are the main subgroup that recognizes and interacts with EVT cells. Since dNK1 can also co-express receptors for CD39 and KIRs, the similarity in the patterns were similar to those of CD39^+^ KIR^+^ eNK cells, suggest that eNK cells may be a population of mature pre-dNK1 cells awaiting pregnancy. In addition, dNK1 cells had more cytotoxic granules, mainly granzyme B and granulysin. However, they responded poorly to stimulation by missing self, which may be related to the high expression of LILRB1. Both dNK1 and dNK2 cells highly express the inhibitory receptor NKG2A and activating receptors NKG2C and NKG2E, while dNK3 cells do not. dNK3 cells express CD103 and respond to nonspecific stimuli significantly, while dNK1 and dNK2 cells do not (21, 85). In a recent study, a class of memory NK cells exhibiting LILRB1^+^ NKG2C^high^ characteristics, similar to dNK1 cells, was found in dNK cells from multiple pregnancies. The percentage of these memory NK cells was low in first pregnancies but rapidly increased in second and subsequent pregnancies. PTdNK cells support vascularization by secreting interferon-gamma (IFN-γ) and vascular endothelial growth factor-alpha (VEGFα) (22). Overall, these findings suggest that dNK cells include three subpopulations that are presumed to be responsible for different functions owing to their different phenotypes. dNK1 cells may play a role in embryo recognition and memory in multiple pregnancies. The three subtypes of dNK cells may interact with other cells in terms of immune regulation and placental development. Further research is needed to confirm these possibilities.

#### 2.3.2 Key Cytokines for dNK Cell Survival and Differentiation

IL-15 is a pleiotropic cytokine that supports NK cell proliferation, differentiation, and development (86, 87). IL-15 production by DSCs during the menstrual cycle and pregnancy is regulated in a spatial and temporal manner. In the late secretory stage, progesterone binds to the progesterone receptor and GATA2 to induce the secretion of IL-15. After embryo implantation, DSCs and macrophages secrete more IL-15, which drives the differentiation and proliferation of uNK cells (88). These findings are supported by results from a mouse model that demonstrated the induction of IL-15 prior to embryo implantation, with a peak in the second trimester along with spiral artery remodeling (89). During early pregnancy, DSCs secrete IL-24, which promotes the differentiation of dNK cells with the expression of multiple inhibitory receptors, immune tolerance, and angiogenic cytokines (90). However, endometrial stromal cells (ESCs) secrete lower levels of IL-24, which may be one of the reasons why dNK cells and eNK cells have different receptor profiles. In the presence of transforming growth factor-beta 1 (TGF-β1), hypoxia, and demethylating agents, PBNK cells are induced to transform into NK cells with characteristics similar to those of dNK cells, with non-cytotoxic and pro-angiogenic properties (41, 42). Furthermore, the local elevation of TGF-β1 in the decidua is a key factor mediating the increase in dNK cell subsets and the regulation of immune function (76). These findings indicate that uNK cells are accurately regulated in spatial and temporal manners by a variety of factors to facilitate normal embryo implantation and successful pregnancy, especially IL-15 and TGF-β1.

#### 2.3.3 dNK Cell Function

##### 2.3.3.1 Pathogen Invasion

In normal pregnancy, the maternal-fetal interface is in a state of immune tolerance. When microorganisms invade, multiple immune pathways at the maternal-fetal interface are activated to maximize the safety of the mother and fetus. Several pathogens are capable of infecting the placenta. The transmissive routes of these pathogens, maternal-fetal defense mechanisms, current treatment status, and future treatment strategies have been comprehensively reviewed in previous literature (91–93). At this point, we will discuss a few instances where the specific interaction of pathogens with uNK cells have been deciphered (**Figure 3A**). For example, when exposed to human cytomegalovirus (HCMV)-infected decidua fibroblasts, dNK cells lose their decidual characteristics by reducing the expression level of CD56 while increasing the expression of CD16. This enhances the cytotoxic ability of dNK cells by significantly increasing the expression of CD94/NKG2C or 2E activating receptors and NKG2D ligands. Overall, the functions of dNK cells prevent further infection of fetal tissues (94). KIR2DS1^+^ dNK cells were demonstrated to be more cytotoxic than KIR2DS1^-^ dNK cells when exposed to HCMV-infected DSCs. dNKs increase their responsiveness to placental HCMV infection through activation of the KIR2DS1/HLA-C2 interaction. This reduces serious pregnancy complications, such as miscarriage and preterm birth (19). Crespo et al. showed that dNK cells can transport the antimicrobial peptide granulysin (GNLY) through nanotubes into placental trophoblast cells to clear intracellular *Listeria monocytogenes* infection. Trophoblast cells are not damaged and the placenta is protected against microbial damage (23). dNK cells can also control human immunodeficiency virus-infected decidual macrophages through cell-to-cell contact and IFN-γ secretion (95). A recent study demonstrated that ZIKV infection of trophoblast cells induces intracellular endoplasmic reticulum stress, which downregulates the expression of HLA-C/G and the ligand of the NK cell surface inhibitory receptor. These events activate dNK cells. The activated cells degranulate and kill ZIKV-infected trophoblast cells, reduce viral transmission, and ensure healthy embryo growth (96). These findings suggest the importance of activated dNK cells in the defense against infection and the spread of placental pathogens. Studies are needed to explore the pathways used by dNK cells to defend against infection by other pathogens during pregnancy, as well as the changes in the functions and characteristics of dNK cells in defense against pathogen infection and induction of maternal-fetal immune tolerance. The findings will clarify the understanding of the functions and related mechanisms of dNK cells during pregnancy.

##### 2.3.3.2 Uterine Spiral Artery Remodeling

During the early stages of human pregnancy, the placenta gradually forms to accommodate oxygen and nutrients required by the embryo. During placenta formation, spiral arteries are transformed into large-capacity, low-resistance thin-walled blood vessels. This change requires the cooperation of a variety of local cells and cytokines. dNK cells are abundant in the vicinity of uterine spiral arteries and may contribute to the remodeling of the uterine spiral artery by regulating the invasive ability and motility of EVT cells and secretion of cytokines (**Figure 3B**). For example, dNK cells can induce the destruction of vascular smooth muscle and the degradation of extracellular matrix by secreting cytokines and angiogenic factors, such as angiopoietin-1 (Ang-1), Ang-2, placental growth factor (PLGF), IFN-γ, VEGFα, and VEGF-C (14, 31). Moreover, dNK cells can also induce the dedifferentiation of spiral artery vascular smooth muscle cells by secreting TGF-β1, Ang-1, and Ang-2, and increase their motility to migrate into the decidua stroma, undergo apoptosis, and become phagocytosed by macrophages (97). In addition, dNK cells can promote EVT recruitment and migration by producing IL-8 and IP-10 (CXCL10), RANTES (CCL5), and leukemia inhibitory factor and can also induce EVT motility by secreting hematopoietic growth factor (14, 98, 99). These findings indicate that cytokines secreted by dNK cells are key factors in arterial remodeling. How the secretory capacity of dNK cells is triggered in the context of the decidua is unclear and needs to be studied.

Several recent studies have demonstrated that the inhibitory receptors of dNK cells and ligands expressed by EVT cells work together to mediate the secretion of various cytokines from dNK cells and participate in arterial remodeling. For example, KIR2DL4, a member of the killer cell KIR family, has roles in activating NK cells and inducing secretion of cytokines and chemokines, although it has a long cytoplasmic tail typical of inhibitory KIRs (100). Rajagopalan et al. reported that KIR2DL4 on dNK cells binds to EVT-expressed non-classic MHC class I molecule HLA-G or secreted sHLA-G. This activates the nuclear factor-kappa B (NF-κB) pathway, which converts dNK cells to a senescence-associated secretory phenotype. This phenotype features the production of large amounts of pro-inflammatory factors, such as TNF-α, IL-1β, and IFN-γ, and pro-angiogenic factors, such as IL-6 and IL-8, to promote angiogenesis and EVT invasion (101, 102). Moreover, KIR2DS4, KIR2DS1, and some alleles of KIR2DS5 in dNK cells can bind to HLA-C, promoting trophoblast cell invasion by secreting GM-CSF (52, 66). Pregnancy-trained memory NK (PTNK) cells that express high levels of NKG2C and LILRB1 and bind specifically to HLA-E and HLA-G on the EVT surface, respectively. These interactions induce the secretion of VEGF and IFN-γ in dNK cells, which promotes remodeling of the uterine spiral arteries (22). Vascular remodeling in the placenta is partly attributed to the synergistic interaction between maternal dNK cells and fetal trophoblast cells, during which ligands on the trophoblast surface trigger dNK cells to produce a series of cytokines and growth factors. In addition, at maternal-fetal interface, multiple receptors and ligands expressed on maternal cells interfere with the secretory function of dNK cells, which have a potential research value (103).

Mouse models have revealed that IFN-γ secreted by uNK cells is an important mediator of uterine spiral artery remodeling (104–106). A more recent study found that IFN-γ expression was significantly reduced in a mouse model of NKG2A^-^ uNK. The vessel wall was significantly thickened, lumen area was normal, and uterine artery remodeling was impaired. These results indicate the necessity of NKG2A for uterine artery remodeling during pregnancy (107). NKG2A is also present on human dNK cells. HLA-E serves as its ligand on the surface of trophoblast cells. Further studies of the NKG2A receptor and HLA-E binding on human dNK cells are needed.

##### 2.3.3.3 Promotion of Fetal Development

**Figure 5:**
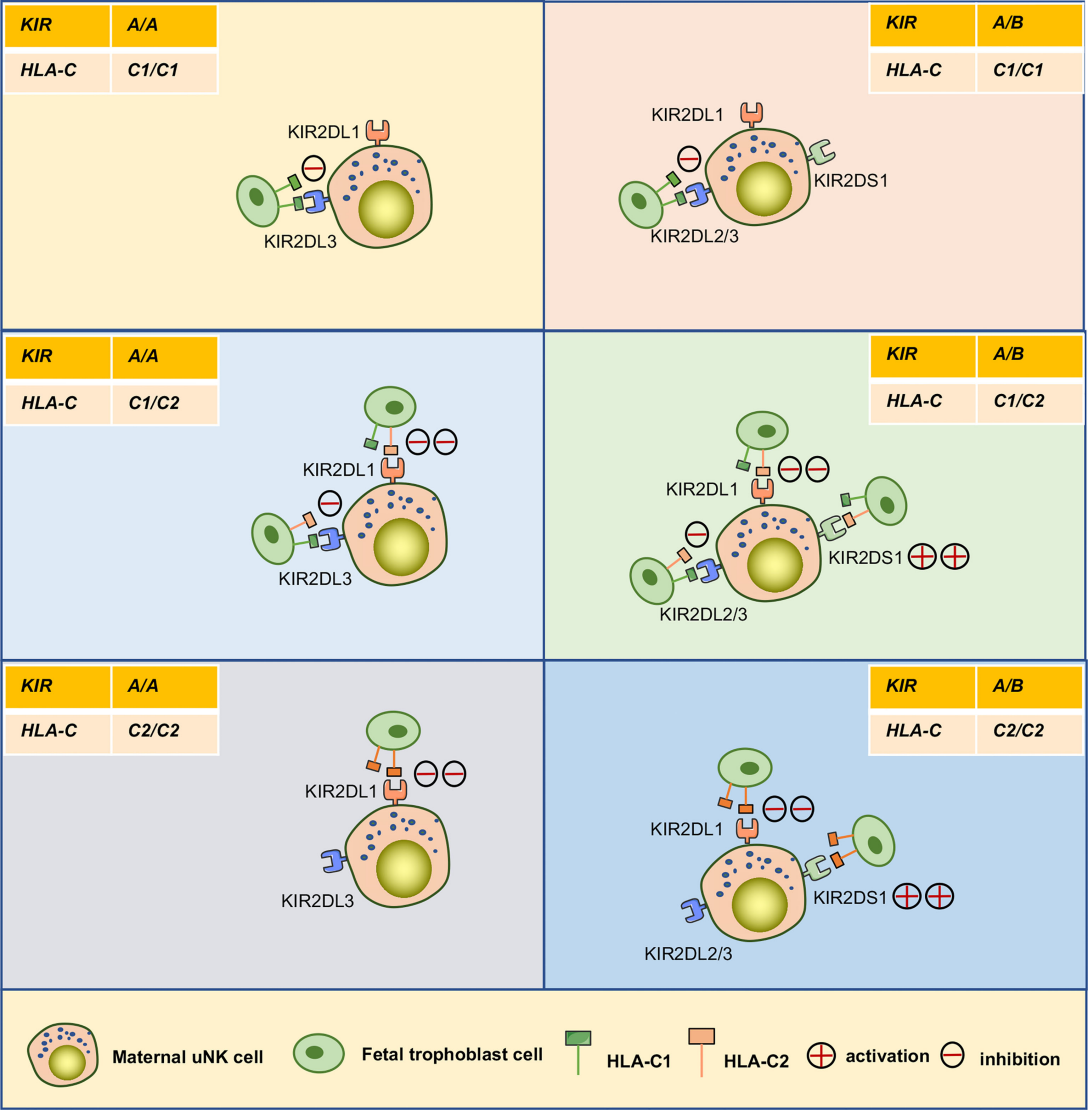
Interaction between KIRs of human maternal uNK cells and HLA-C of fetal trophoblast cells. The interaction of the mother’s two *KIR* genotypes (*A/A*, *A/B*) and the embryo’s three possible *HLA-C* genotypes (*C1/C1*, *C1/C2*, *C2/C2*) was analyzed. The *C2* gene in the figure was at least one from the father. *KIR A* haplotypes mainly feature inhibiting receptors, including KIR2DL1and KIR2DL3. The *KIR B* haplotypes mainly include KIR2DL1, KIR2DL2, and activating receptor KIR2DS1.

Access to maternal-fetal interface, dNK cells participate in the remodeling process of the uterine spiral arteries that providing adequate nutrients and oxygen for the fetal development. These cells also promote embryonic development in other ways (**Figure 3C**). Recently, Fu et al. found that CD49a^+^ Eomes^+^ NK cell subpopulations in humans and mice can secrete growth-promoting factors (GPFs), including pleiotrophin (PTN), osteoglycin (OGN), and osteopontin (OPN), in response to the interaction of HLA-G and immunoglobulin-like transcript 2 (ILT2) to promote embryonic development (20). Additionally, the frequencies of CD49a^+^uNK cells and expression of GPFs were decreased in aged mice. Strikingly, transferring induced CD49a^+^uterus-like NK cells reversed FGR outcome in NK cell-deficient transgenic mouse modes or aged mice (20). Furthermore, they found that the interaction between HLA-G and ILT2 induces the expression of PBX1 by mediating activation of the protein kinase B, which induces the secretion of GPFs (108). However, it is still uncertain whether uNK cells are the main source of GPFs and the specific ways in which GPFs affect embryonic growth (109). The combination of KIR and HLA-C ligands contributes to successful reproduction by maintaining birth weight between the two extremes. In general, increased expression of maternal inhibitory KIRs on uNK cells are associated with low-weight embryos, whereas increased expression of activating KIRs are associated with larger embryos (78, 110). For example, the combination of KIR2DL1 and C2 can induce a strong inhibitory signal and is more specific than the C1/C2 combination with KIR2DL2/3. When activator KIR2DS1 is bound to the fetal HLA-C2 ligand, it promotes an increase in fetal weight and placental development (78). A maternal *KIR AA* genotype lacking the activator KIR2DS1 can more easily lead to a low-weight fetus. However, when the embryo is homozygous for *HLA-C1*, there is no effect of any *KIR* genotype on any clinical outcome during pregnancy (110) (**Figure 5**).

As recently reported, all NK cells expressed the inhibitory receptor for human KIR2DL1 in transgenic female mice, and their embryonic growth was significantly restricted only in mating males that also expressed human HLA-C (111). Another mouse study showed that maternal NKG2A is involved in the education of uNK cells and is also required for optimal fetal growth. In particular, embryonic growth is significantly reduced, and the frequency of abnormal brain development in mothers with knockout of the NKG2A receptor is similar to that of NK cell deficiency or hypofunction (107). The above results demonstrate that KIR/HLA-C and NKG2A regulate fetal growth and brain development. The collective findings also illustrate the importance of the diversity of human KIRs and HLA-C, with different combinations of KIRs and HLA-C appearing in each pregnancy to ensure reproductive stability in the entire community.

##### 2.3.3.4 Induction of Immune Tolerance

The establishment of the immune system during pregnancy is complicated by the fact that the embryo inherits half of its genes from the father. Unraveling the mechanisms underlying immune tolerance during pregnancy has been the goal of several studies. dNK cells have become a focus of research because of their frequency at the maternal-fetal interface, specific phenotype, and ability to identify trophoblast cells (**Figure 3D**). Under quiescent circumstances, dNK cells are less cytotoxic even though they contain more cytotoxic granzymes. The inability of dNK cells to polarize microtubule-organizing centers and perforin-containing granules to synapses is one of the reasons for their lack of cytotoxicity (13). The Gal-9/Tim-3 signaling pathway may be involved in the hindrance of degranulation (112). NK cell cytotoxicity is determined by the balance between inhibitory and activating signals (13). Changes in inhibitory or activating receptors on the surface of dNK cells may be responsible for their reduced cytotoxicity. For example, NCRs (NKp46, NKp30, and NKp44) are involved in the maintenance of PBNK cell cytotoxicity, although PBNK cells in the resting state do not express NKp44, whereas dNK cells can express three NCRs. Interestingly, NCRs show distinct functions in dNK cells. For example, NKp44 reportedly displayed an inhibitory ability, whereas NKp30 promoted cytokine secretion, and only NKp46 could activate dNK cells (77). However, although freshly isolated dNK cells showed significant cytotoxicity upon activation of NKp46, they were significantly affected when co-expressed with the inhibitory receptor NKG2A (113). Moreover, the interaction of early pregnancy trophoblast cells with dNK cells reduced the expression of NKG2D and NKp80, resulting in the functional inhibition of dNK cells (60). In addition, 2B4 is widely expressed in different leukocyte populations, and its ligand CD48 can mediate opposite functions. In mature NK cells, 2B4 can cooperate with NCRs or NKG2D to mediate optimal NK cell activation and function as a coreceptor (114). However, 2B4 can also transmit inhibitory signals to immature NK cells derived from differentiated CD34+ stem cells *in vitro* (115). Binding of the dNK cell inhibitor receptor 2B4 to CD48+ cells lead to inhibition of cytolytic activity and IFN-γ secretion (116). It has been shown that PBNK cells may lose their cytotoxic ability by acquiring HLA-G, a non-classical MHC class I molecule, from transfected melanoma cell lines through trogocytosis, while the cytotoxic ability can be reversed once the phagocytosed HLA-G is degraded or turned over. This immune tolerance mechanism also exists in dNK cells (117, 118). HLA-G is expressed on the surface of EVT cells, which is a ligand for LILRB1 and KIR2DL4 in dNK cells. The HLA-G cycle can also control the cytotoxicity of dNK cells. In this process, dNK cells lose the cytotoxic ability *via* endocytosis HLA-G from HLA-G^+^EVT cells in a contact dependent manner. When acquired HLA-G is degraded in response to IL-15, the cytotoxicity of dNK cells recover (119). Multiple receptor-ligand interactions control dNK cytotoxicity and pro-inflammatory responses *in vivo*. The expression and function of dNK cell receptors result in corresponding changes in the adaptation to the specific environment of the decidua, which is one of the mechanisms of pregnancy immune tolerance. However, whether other NKRs in dNK cells undergo corresponding changes requires further study. In addition, whether other immune cells in the decidua also undergo corresponding changes needs to be determined.

In addition to the immune tolerance function induced by the aforementioned dNK activating and inhibitory receptors, dNK cells can also induce immune tolerance through other receptors on their surface interact with other immune cells. CD39 is a member of the ENTPD family. CD39 is expressed on the surface of a variety of lymphocyte populations, including B cells, DC, neutrophils, monocytes, and some NK and T cells. CD39 combined with CD73 degrades extracellular ATP to extracellular adenosine, which has significant immunosuppressive effects and the ability to limit the spread of inflammation (120). CD39 is highly expressed on dNK cells and can bind to CD73 on the surface of epithelial glands and EVT cells to convert ATP to adenosine to prevent immune activation (21).

## 3 uNK Cells Under Pathological Conditions

### 3.1 Recurrent Miscarriage

**Figure 6:**
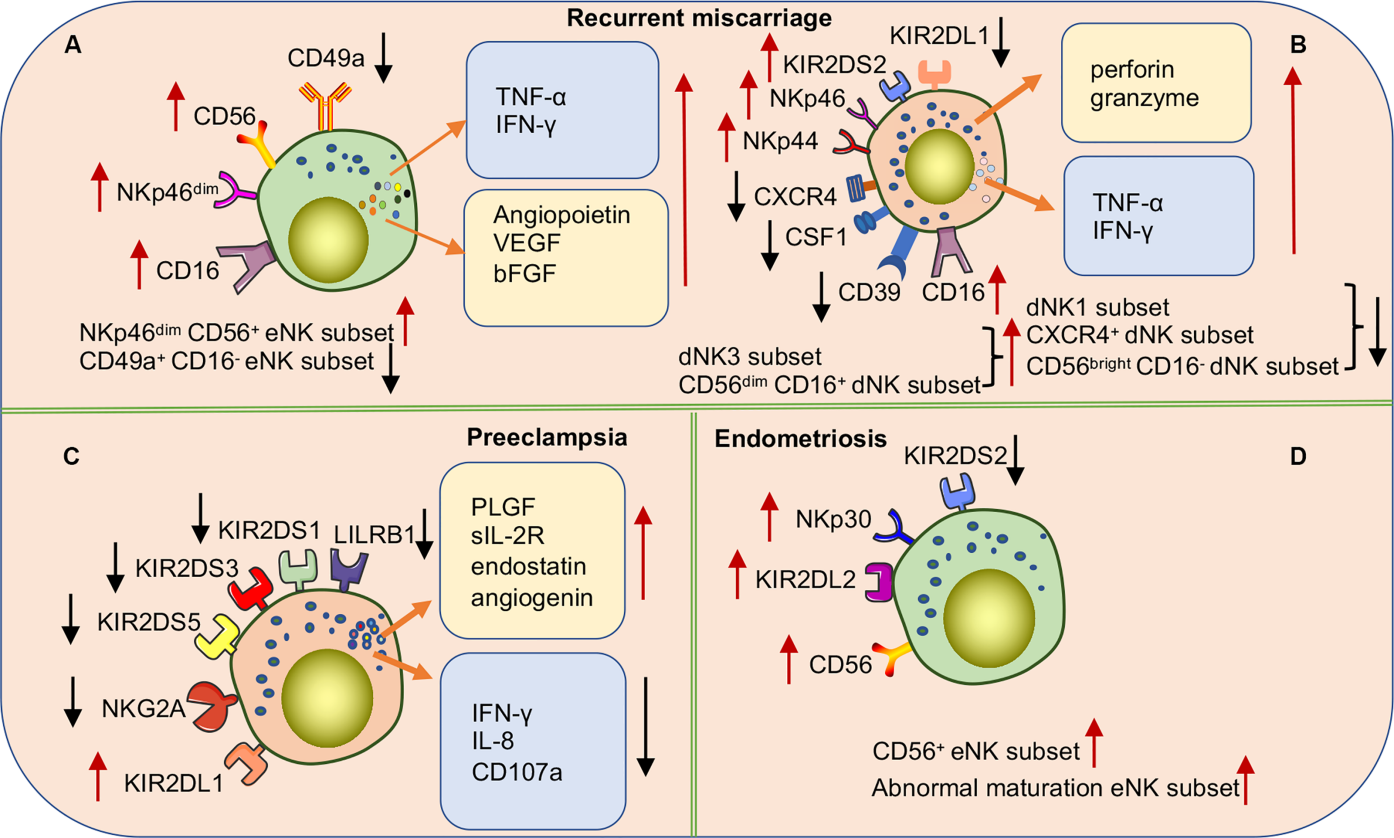
Subpopulations or functional alterations of human uNK cells in pregnancy complications. **(A)** NKp46^dim^ CD56^+^ eNK cell populations in RM patients are significantly increased while the CD49a^+^ CD16^-^ eNK cell populations significantly decrease. In addition, there are a significant accumulation of various cytokines in eNK cells. **(B)** In women with RM, the expression of various NCRs and activating receptor KIR2DS2 is up-regulated, and the inhibitory receptor KIR2DL1 is down-regulated to activate dNK cells. The frequencies of dNK1 cell, CXCR4^+^ dNK cell, and CD56^bright^ CD16^-^ dNK cell subsets are significantly decreased while dNK3 cells and CD56^dim^ CD16^+^dNK cell subsets significantly increased. **(C)** The cytokine secretion profile of dNK cells is markedly altered in preeclampsia patients. Downregulation of activating receptor expression on the surface of NK cells or upregulation of inhibitory receptors inhibits NK cell activation causing a high risk of developing PE. Especially when maternal NK cells carrying the KIR AA genotype are combined with embryos carrying the HLA-C2 gene. **(D)** In women with endometriosis, higher frequencies of CD56^+^eNK cells and abnormal maturation eNK cells are found in eutopic endometrium. Upregulation of inhibitory receptors or downregulation of activating receptors leads to over suppression of eNK cell function associated with the occurrence and severity of endometriosis.

Female infertility has long been a challenge for obstetric reproductive disorders. Although embryo transfer causes infertility, RM still occurs in some patients, even in healthy embryos, suggesting that pathological changes in the endometrium cause pregnancy failure. Because eNK cells participate in the decidualization process of the endometrium, a series of studies explored the correlation between eNK cells and RM (**Figure 6A**). Increasing evidence suggests that the frequency of eNK cells is elevated in patients with RM (121–124). Multiple studies have shown that eNK cell frequency in patients with RM induces increased vascularization of the preimplantation spiral artery and defective vascular transformation, leading to opening of the early maternal-fetal circulation. These effects can cause excessive oxidative stress, leading to pregnancy failure (72, 123). The results of the study of Chen et al. also supported the above point of view; they found that the expressions of angiopoietin, VEGF-A, and basic fibroblast growth factor (bFGF) were significantly higher in eNK cells of women with RM than in normal reproductive females (125). In addition, multiple studies have shown that increased numbers of CD56^+^ CD16^+^ uNK cells lead to an inflammatory environment during implantation or late decidualization, which is a high-risk factor for RM (126, 127). The correlation between NKp46 activating receptor expression in NK cells and RM is controversial. Several studies have demonstrated that higher proportions of NKp46^+^ CD56^+^ eNK cell populations may result in a greater risk of infertility (126). However, recent reports suggest that low expression of the NKp46 receptor on eNK cells is more common in women at high-risk for RM (70, 128). In one study, NKp46^+^ eNK cells were classified into low-expression (NKp46^dim^ eNK) and high-expression (NKp46^bright^ eNK) groups according to different fluorescence intensities. NKp46^dim^ eNK cells were reportedly involved in cell killing and NKp46^bright^ eNK cells in cytokine production, demonstrating that these two clusters play different roles in the immune regulation of the endometrium (129). Further studies are needed to verify the relevance of NKp46 in RM, and consistent criteria need to be applied. In addition, a recent study found an altered cytokine secretion profile of eNK cells in women with RM. Unlike in women with normal fertility, the TNF-α/IL-4, IFN-γ/IL4, TNF-α/IL-10, and IFN-γ/IL10 ratios in eNK cells were significantly elevated in women with RM (128). The results indicate the association of changes in the receptor and cytokine profiles of eNK cells with RM. However, the causal relationship between RM and eNK cells needs to be reconsidered in future studies, as almost all analyses of eNK cells have been retrospectively conducted in patients after RM, rather than in prospective trials. Considering the difficulty of obtaining samples, menstrual blood may be the best choice for prospective experiments. Recently, Tong et al. found that CD49a^+^ NK cells in menstrual blood are a more accurate and sensitive indicator of the endometrial status. The authors also found that changes in CD49a^+^ EOMES^+^ NK, CD56^+^ CD49a^+^ NK, or CD49a^+^ CD16^-^ NK in menstrual blood were better predictors of RM (25). Therefore, a prospective study of NK cells in menstrual blood would provide comprehensive data of the pathogenic mechanism of eNK in RM and also provide a new direction for predicting the risk of RM.

In addition, studies on dNK cells in patients with RM have also made progress (**Figure 6B**). For example, multiple studies have shown that the frequency of CD56^+^ CD16^-^ dNK cells is decreased in the decidua of women with RM, whereas the frequency of CD56^+^ CD16^+^ dNK cells is significantly increased. Moreover, the proportion of CD56^+^ CD16^-^ dNK and CD56^+^ CD16^+^ dNK cells highly expressing NKp44 and NKp46 receptors was reportedly significantly increased, which increased the cytotoxicity of lysed target cells (130–132). In addition, compared to normal reproductive women, dNK cells isolated from patients with RM *in vitro* were found to secrete higher levels of TNF-α, hindering endometrial decidualization (133). Furthermore, a recent study demonstrated that induced decidual NK cells (idNKs) were generated by *in vitro* culture of PBNK cells under hypoxia, TGF-β1, IL-15, and 5-aza-2-deoxycytidine, with phenotypes and functions similar to those of dNK cells. The authors also described that impaired secretory VEGF function in idNK cells and altered expression of KIR2DL1 and ILT-2 receptors may be associated with RMs (134). Recently, with an in-depth study of dNK cells, new dNK cell subsets have been identified and proven to be associated with the occurrence of RM. Tao et al. identified a new dNK cell subset (CXCR4^+^ CD56^bright^), recruited from peripheral blood to the decidua by trophoblast cells. CXCR4^+^ CD56^bright^ dNK cells secrete large amounts of IL-4 and maintain pregnancy immune tolerance by inducing Th2 bias in normal pregnant women. In contrast, patients with RM have significantly reduced numbers of CXCR4^+^ CD56^bright^ dNK cells and impaired ability to induce Th2 differentiation (135). Chen et al. also identified a novel NK cell subset (CSF1^+^ CD59^+^ KIR^+^) in the normal decidua and demonstrated that this subset is significantly reduced in the decidua of patients with RM (136). In addition, multiple studies have investigated the heterogeneity of immune cells in the decidua of RM patients using single-cell RNA sequencing (scRNA-seq). Sequencing results showed that RM patients had a lower frequency of dNK1 cells and higher proportions of dNK2 and dNK3 cells than women with normal pregnancies (29). Mass spectrometry results demonstrated that the number of CD11c^high^ NK cells with pro-inflammatory effects was significantly increased in chromosomally normal women with RM. Given that CD11c^high^ NK and dNK3 do not express CD39 and that both have pro-inflammatory properties, the function of CD11c may be similar to that of dNK3 (137). Another recent study found a significant decrease in the proportion of CD39^+^ CD18^-^ dNK(dNK1) cells in the decidua of patients with RM (29). The collective findings suggest that subpopulations, cytokines, and receptors of dNK cells in patients with RM may be important mechanisms of pathogenesis. The findings also improve our understanding of dNK cell heterogeneity at the maternal-fetal interface in disease states and provide clues for future treatments of RMs targeting dNK cells. Interestingly, specific KIRs and embryonic HLA ligands in women with RM increase susceptibility to RM. Faridi et al. observed that the *KIR* gene *BB* genotype is more common in RMs, and that multiple activated *KIR* genes are high-risk factors for pathogenicity (138). These results suggest that the pathogenesis of RM may be related to excessive activation of uNK cells caused by the increased expression of maternal-activating KIRs or a decrease in inhibitory KIR receptors. The number of partners expressing both C1 and *KIR2DS2* is increased in patients with RM. However, the number of partners expressing both C2 and *KIR2DL1* is significantly lower than that in the control group (139). Therefore, specific KIR and HLA-C genotypes may be used to predict reproductive outcomes in women with RM.

### 3.2 Preeclampsia and Fetal Growth Restriction

PE is a serious pregnancy complication and a leading cause of maternal and perinatal morbidity and mortality worldwide. It is closely related to FGR. The primary placental pathology of PE is inadequate infiltration of EVT and impaired spiral artery remodeling. The typical symptoms are hypertension and proteinuria, which can develop into multisystemic impairment (140). Its pathogenesis is unclear. However, several studies have shown that immune imbalance during pregnancy is one of the key causes of PE. During physiological pregnancy, dNK cells can promote trophoblast cell invasion, uterine spiral artery remodeling, and embryonic development. Therefore, the occurrence of PE may be related to changes in the subpopulation of dNK cells or impaired function (**Figure 6C**) (141). The changes in the proportion and number of dNK cells in patients with preeclampsia are contentious. Different studies have observed different changes in the frequency of dNK cells in patients with PE or FGR compared to normal pregnancies (142–146). This may be caused by the fact that the physiological onset of PE likely occurs very early during pregnancy, ahead of the appearance of clinical symptoms, making it difficult to obtain qualified samples for precise examination. The method used in the detection and severity of the disease may also have affected the results of the experiments.

Doppler ultrasound scans can be used to measure the uterine artery flow resistance index (RI), which reflects the level of spiral arterial remodeling. A high RI is a surrogate indicator of the degree of poor spiral arterial remodeling. It was found that PLGF, soluble IL-2 receptor, endostatin, and angiogenin secreted by dNK cells in high RI samples of early termination of pregnancy significantly increased, and angiopoietin and endostatin altered trophoblast functions (147). In addition, the surface receptors KIR2DL/S1,3,5 and LILRB1 were significantly reduced in dNK cells isolated from patients with an elevated uterine artery RI. After blocking the LILRB1 receptor *in vitro*, the expression of CXCL10 and TNF-α can be altered to modulate the invasive ability of EVT (53). In addition, compared with normal pregnancy, the expression of soluble HLA-G was significantly decreased in serum and placenta of patients with PE (148–151). Furthermore, bioinformatic analyses revealed impaired maturation of the endometrium and uNK cells during the endometrial secretory phase and early pregnancy in women who developed PE (152). Decidual TGF-β has been mentioned as an important cytokine for the proliferation and differentiation of dNK subsets. Changes in the level of local TGF-β secretion in the decidua are thought to mediate impaired maturation of dNK cells. Elevated TGF-β in the decidua of women with PE inhibits the activation of dNK cells by downregulating the expression levels of IFN-γ, IL-8, and CD107a, leading to uteroplacental pathology associated with preeclamptic seizures (76). These results indicate that the receptors, cytokines, and functions of dNK cells are altered in the PE disease state.

Previous population-based studies suggest that genetics from both parents have a mild correlation with the development of PE (153–155). Subsequent studies extended epidemiological findings by demonstrating that the genetic combination between the maternal *KIR* genotype and the fetal *HLA-C* genotype is associated with predisposition to PE (83). For example, several studies have shown that specific combinations of dNK cell KIR receptors with HLA-C on the surface of EVT increase the risk of PE, such as the combination of the maternal *KIR AA* gene and chimeric paternal *HLA-C2* genotype in embryos. C2 binds more tightly to KIR2DL1 and can strongly inhibit dNK cell function, affecting uterine artery remodeling and causing placental structural abnormalities (83). When a pregnant mother has the *KIR B* genotype and the embryo has the *HLA-C2* combination, KIR2DS1^+^ uNK cells can be activated to secrete GM-CSF to enhance trophoblast invasion and uterine artery remodeling, resulting in a healthy pregnancy. In contrast, when pregnant mothers with the *KIR AA* gene inhibit uNK cell activation and reduce cytokine secretion, the risk of PE increases (66). These results may have some clinical implications; For instance, C2/C2 donor sperm will confer the trophoblast cells with at least one allele of HLA-C2 expression, resulting in an increased maternal risk of PE compared to the C1/C1 sperm donors (16). Of note, the influence of HLA-C/KIR genetic combination might be affected by ethnic background. For example, the inverse association between KIR AA genotype and HLA-C2 in a historically isolated population, such as Japanese and the Australian Aboriginals, was greater than the ones in countries like the UK, and Afro-Caribbean, which resident with diverse ethnic groups (83). Several studies have shown that the *KIR* genotype in Europeans features a higher frequency of the *KIR2DS1* gene and a lower *HLA-C2* gene than in Africans (78, 80, 156). Another report suggested that Ethiopians also have a high frequency of the protective *KIR2DS1* gene, unlike other East African populations (156). Shreeve et al. showed that NKG2A inhibitory receptors could educate uterine NK cells and promote vascular remodeling, fetal growth, and embryonic brain development. Importantly, this study also showed that HLA-B variants are associated with preeclampsia, and a genome-wide analysis of more than 7,000 preeclampsia cases revealed a 7% increased risk of PE when the *HLA-B* allele was unfavorable for NKG2A education. These results suggest that the maternal HLA-B→HLA-E→NKG2A pathway optimizes pregnancy outcome (107). These results reveal a relationship between the genetics of both parents and the risk of PE.

### 3.3 Endometriosis

Endometriosis is a hormone-dependent, slow-moving inflammatory process whose pathology is characterized primarily by ectopic endometrium beyond its normal location, commonly in pelvic structures. The primary symptoms include dysmenorrhea, chronic pelvic pain, and infertility. The theory of retrograde menstruation is now generally accepted. Almost all women have experienced retrograde menstruation at some point in their lifetimes (157), but only a few will have symptoms of endometriosis, which may be influenced by other factors. The immune system is thought to be important in its pathophysiology and symptomatology, especially uNK cells (**Figure 6D**). Owing to the prevalence of retrograde menstruation, analysis of changes in the cellular composition of immune cells in menstrual blood may become an important tool for studying endometriosis. The frequency of MBNK cells in women with endometriosis was significantly lower than that in healthy women, and the phenotypes remained similar. uNK cells participate in decidualization by targeting the clearance of senescent DSCs, whereas the reduced frequency of uNK cells in menstrual blood in endometriosis impairs the clearance of senescent DSCs and influences the decidualization process, which acts as a factor in the pathogenesis of endometriosis (33, 158). Multiple studies have demonstrated that the proportion of CD56^+^ or CD16^+^ NK cells in endometriotic tissue is significantly reduced compared to that in healthy women and does not exhibit typical uNK cells similar to eutopic endometrium characteristics (159, 160). Data from single-cell sequencing of the eutopic endometrium in patients with endometriosis and from healthy women suggest that reduced NK cell numbers in endometriotic lesions and the impaired ability of NK cells to clear endometriotic lesions promote the development of endometriosis (161). Transcriptome analysis of proliferative and early secretory samples from endometriosis and healthy women has revealed increased frequencies of CD56+ NK and CD8+ T subsets in ectopic endometriosis and significantly reduced frequencies of CD163+ macrophages. In addition, the tumor necrosis factor, IL-17, and mitogen-activated protein kinase signaling pathways were enriched in the eutopic endometrium of patients with endometriosis, revealing a pro-inflammatory feature in the endometrial immune environment (162). Interestingly, in the ectopic endometrium in endometriosis, uNK cells increase gradually from the proliferative stage and reach their highest level during the anaphase of secretion. This trend is consistent with that of the average person (126, 160). Together, these results suggest that uNK cells from endometriosis have increased numbers and an impaired ability to clear lesions. However, the increasing trends of uNK cells in the eutopic endometrium are consistent with those in healthy humans. Investigation of the possibility that some uncertain factors at the lesion site result in the abnormality of NK cells may be a breakthrough for the treatment of endometriosis.

Women with endometriosis have lower endometrial stem cell factor levels, leading to abnormal maturation of the local uNK cell population, which affects embryo implantation and leads to endometriosis-related infertility (163). Furthermore, women with endometriosis have lower uNK activity than normal women; however, uNK cytotoxic activity increases when patients with endometriosis experience infertility and/or RMs (47). The interaction between macrophages and ESCs induces IL-10 and TGF-β secretion, which reduces uNK cytotoxicity. This triggers immune escape from ectopic foci, leading to further development of endometriosis (164). It has also been reported that endometriosis is related to changes in NK cell NCRs and activating receptors. Drury et al. found that NKp30 and its ligand, BAG6, were significantly upregulated in NK cells in the eutopic endometrium of women with endometriosis (160). However, the expression of NKp30 and NKG2D in CD56^+^ NK cells decreased in the peritoneal fluid. The expression of NKG2D ligands in eutopic and ectopic endometria of endometriosis was also significantly lower than that in healthy individuals. These findings suggest that changes in NCRs and NKG2D expressed on NK cells of peritoneal fluid and NKG2D ligands expressed on endometrial cells may be complex mechanisms by which endometriotic lesions escape immune clearance (165). In support of the above results, González et al. found that the peritoneal fluid of endometrial patients had higher levels of soluble NKG2D ligands than healthy individuals, especially in patients with deep invasive endometriosis. The shedding of NKG2D ligands from uNK cells in the endometrium is responsible for these results (166). HLA-G may be associated with the development of endometriosis, as it is expressed in both the *in situ* and ectopic endometria of patients with endometriosis and expressed at abnormally high levels in peritoneal endometriotic lesions (167). In addition, HLA-G was expressed in both *in situ* and ectopic endometria of patients with adenomyosis, and HLA-G expression was also detected in ectopic endometrial lesions during menstruation. The findings suggest that epithelial cells carrying HLA-G may enter the peritoneal cavity during retrograde menstruation, allowing a local reaction of antigen with KIR2DL4 and mediating immune escape (168–170). A retrospective study showed that polymorphisms in *HLA-G*, *LILRB1*, and *LILRB2* genes contributed to the susceptibility and severity of endometriosis, but not *KIR2DL4* polymorphisms (171). Other studies have suggested that the *KIR2DL2* gene is associated with deep endometriosis through the inhibition of NK cytotoxic activity and impaired clearance of ectopic endometrial cells (172). Moreover, the *KIR* and *HLA-C* genotypes have also been associated with the pathogenesis of endometriosis. The number of KIR2DS2-positive individuals with endometriosis was reduced in a Han Chinese population (173). The above results suggest that endometriosis may be induced by the shedding of activating receptors or reduced ligand expression in NK cells. Changes in other inhibitory receptors and their ligands might also be responsible for this phenomenon.

## 4 Potential of uNK Cells in Treating Reproductive Diseases

In early studies, it was widely believed that uNK cells are detrimental to pregnancy success (174–178). Thus, multiple tentative immunotherapeutic approaches/drugs designed to reduce the activity of NK cells, such as lymphocyte immunotherapy (LIT), intralipid therapy and injection of tumor necrosis factor alpha (TNFα) inhibitors/immunoglobulins (IVIg)/steroids/glucocorticoids/G-CSF, have been applied to treat RM or recurrent implantation failure (RIF) clinically, which have been comprehensively reviewed and critically analyzed in previous literatures (179–181). In general, there is no uniform evidence that the above-mentioned immunotherapeutic approaches benefit clinical outcomes in patients; and there is no consensus on the role of uNK cells in RM or RIF (180). However, recent studies have shown that uNK cells have special physiological functions in reproduction, especially in the establishment and maintenance of pregnancy (182, 183), and corticosteroids/intralipid therapy that suppress NK cells activity may exacerbate pregnancy failure (184, 185). Additionally, the state of the endometrium or the risk of pregnancy disease may be predicted by the examination of uNK cells in menstrual blood (25). Interestingly, adoptive transfer of *in vitro* induced human uNK like cells to lead to the promotion of mouse fetal growth, suggesting immunotherapy by functional modulation or adoptive transfer of dNK cells might be a potential approach to treat reproductive diseases in future (26).

A conceptual experiment on immunotherapy for pregnancy complications by dNK cell transfer was first performed in a mouse model. The significant reduction in CXCR4^+^ dNK cells was reportedly associated with the occurrence of RM in humans, and embryo resorption rates were reportedly significantly increased in NK-deficient (Nfil3(-/-)) mice compared with those in normal pregnant mice. However, adoptive transfer of CXCR4+ dNK cells markedly reversed pregnancy outcomes in Nfil3(-/-) mice, validating the potential of dNK adoptive therapy (135). Fu et al. also showed that the number of CD49a^+^ Eomes^+^ trNK cells decreased significantly, and the secretion function of growth-promoting growth factors was impaired in patients with RM. Adoptive transfer of CD49a^+^ uNK cells ameliorated the adverse outcomes of embryos in *Nfil3* ^–/–^ pregnant mice (20).

Considering the potential of uNK cells in treating reproductive diseases, in a recent study, hematopoietic stem cells from cord blood, bone marrow, and peripheral blood were successfully induced into functional human CD49a^+^ NK cells (iNK) by three *in vitro*, feeder-free induction systems. It has been demonstrated that iNKs are phenotypically and functionally similar to dNK cells and express growth-promoting and pro-angiogenic factors *in vitro*. Furthermore, these iNK cells can promote fetal growth and improve uterine arterial blood flow in pregnant NCG mice (26). Notably, the latest *in vivo* research shows that human uNK cells can promote the growth and development of mouse embryos, which may be a new approach for understanding the immunotherapeutic effect of human uNK cells. Notably, since dNK cells possess distinct subpopulations with distinct characteristics and functions (85), selection of a right subpopulation uNK cells for transfer/modulating therapy might benefit the outcome. In a recent report, the frequency of dNK1 cells was found significantly lower in patients with RM compared with healthy women while the frequency of dNK3 increased (29), suggesting that the balance control between different dNK cell subsets would be considered for RM treatment. In addition, as a potential predictor for RM and potent function for maintenance of pregnancy (immune modulation/vessel remodel) (21, 24, 25, 29), CD39^+^ KIR^+^ NK cells might also be a target for cell expansion and adoptive transfer.

## 5 Conclusion and Prospects

uNK cells have emerged as key immune cells involved in the physiology and pathology of both non-pregnant and pregnant uteri. The unique microenvironment of the uterus shapes the specific phenotype of uNK cells, which is distinct from NK cells in peripheral organs. After embryo implantation, uNK cells contribute to pregnancy success through phenotype transition and altered receptor/cytokine profiles, resistance to microbial infection, remodeling of uterine spiral arteries, immune tolerance, and fetal development. In contrast, functional impairment or dysregulation of uNK cells may be involved in a variety of reproductive diseases, such as recurrent abortion, preeclampsia, and endometriosis. Owing to their pivotal role in pregnancy immunology, it is expected that uNK cells may be important cellular immunotherapy targets as well as a predictive index for pregnancy complications in the future.

## Author Contributions

MX designed this review. YL, YM, PX, and YY performed the paper selection. MX, ZH, SD, and JH wrote the manuscript. All authors approved it for publication.

## Funding

This work was supported by National Natural Science Foundation of China (81870091and 82001759) and the Science Development Project of Jilin Province (20190201295JC and 20200703012ZP).

## Conflict of Interest

The authors declare that the research was conducted in the absence of any commercial or financial relationships that could be construed as a potential conflict of interest.

## Publisher’s Note

All claims expressed in this article are solely those of the authors and do not necessarily represent those of their affiliated organizations, or those of the publisher, the editors and the reviewers. Any product that may be evaluated in this article, or claim that may be made by its manufacturer, is not guaranteed or endorsed by the publisher.
